# The contribution of human conflict to the development of antimicrobial resistance

**DOI:** 10.1038/s43856-023-00386-7

**Published:** 2023-10-25

**Authors:** Scott J. C. Pallett, Sara E. Boyd, Matthew K. O’Shea, Jessica Martin, David R. Jenkins, Emma J. Hutley

**Affiliations:** 1 Centre of Defence Pathology, Royal Centre for Defence Medicine, Queen Elizabeth Hospital, Birmingham, UK; 2 Antimicrobial Pharmacodynamics and Therapeutics, Department of Molecular and Clinical Pharmacology, University of Liverpool, Liverpool, L69 3GE UK; 3 Department of Clinical Microbiology, Leeds Teaching Hospitals NHS Trust, Leeds, LS1 3EX UK; 4 Department of Clinical Microbiology, University Hospitals of Leicester NHS Trust, Infirmary Square, Leicester, LE1 5WW UK

**Keywords:** Laboratory techniques and procedures, Population screening, Antimicrobial resistance, Clinical microbiology

## Abstract

Pallett et al. discuss the impact of human conflict on development of antimicrobial resistance. They overview approaches to limit the spread of antimicrobial resistance, using the ongoing conflict in Ukraine as an example of the challenges and opportunities.

## Teaser

Human conflict, such as the ongoing conflict in Ukraine, is an important driver of antimicrobial resistance. Here, we describe the challenges when addressing this problem during an ongoing conflict and the opportunities available to reduce the spread of antimicrobial resistance.

## Background

The risk of infection of conflict-related wounds, and the vital importance of strict hygiene measures to reduce the occurrence and consequences, are well known^1^. Antimicrobial resistance (AMR) develops when bacteria, viruses, fungi and parasites mutate, or acquire genetic material from other organisms,  and no longer respond to treatments, eventually becoming multi-drug resistant organisms (MDROs). AMR is one of the most significant global health concerns^2^. Conflict contributes to this global AMR problem because AMR occurs both in the areas where conflict occurs, and also in the areas that those who have been involved in the conflict travel to.

Independent of conflict, the risk of returning travellers arriving home with infected wounds (requiring treatment) or colonised with a MDRO and hence requiring infection prevention and control management (IPC) has led to national guidelines to actively screen people who access healthcare soon after travel^3^. Knowledge of MDRO epidemiology in the countries visited helps healthcare providers to prevent the consequences of infection early^3^. While travel for any reason can lead to infection, medical tourism has been particularly associated with a risk for MDRO transmission^4^, as has travel from conflict areas. Travel from conflict areas can present particular issues, as detailed in this article, especially for those who have been wounded.

**Fig. 1:**
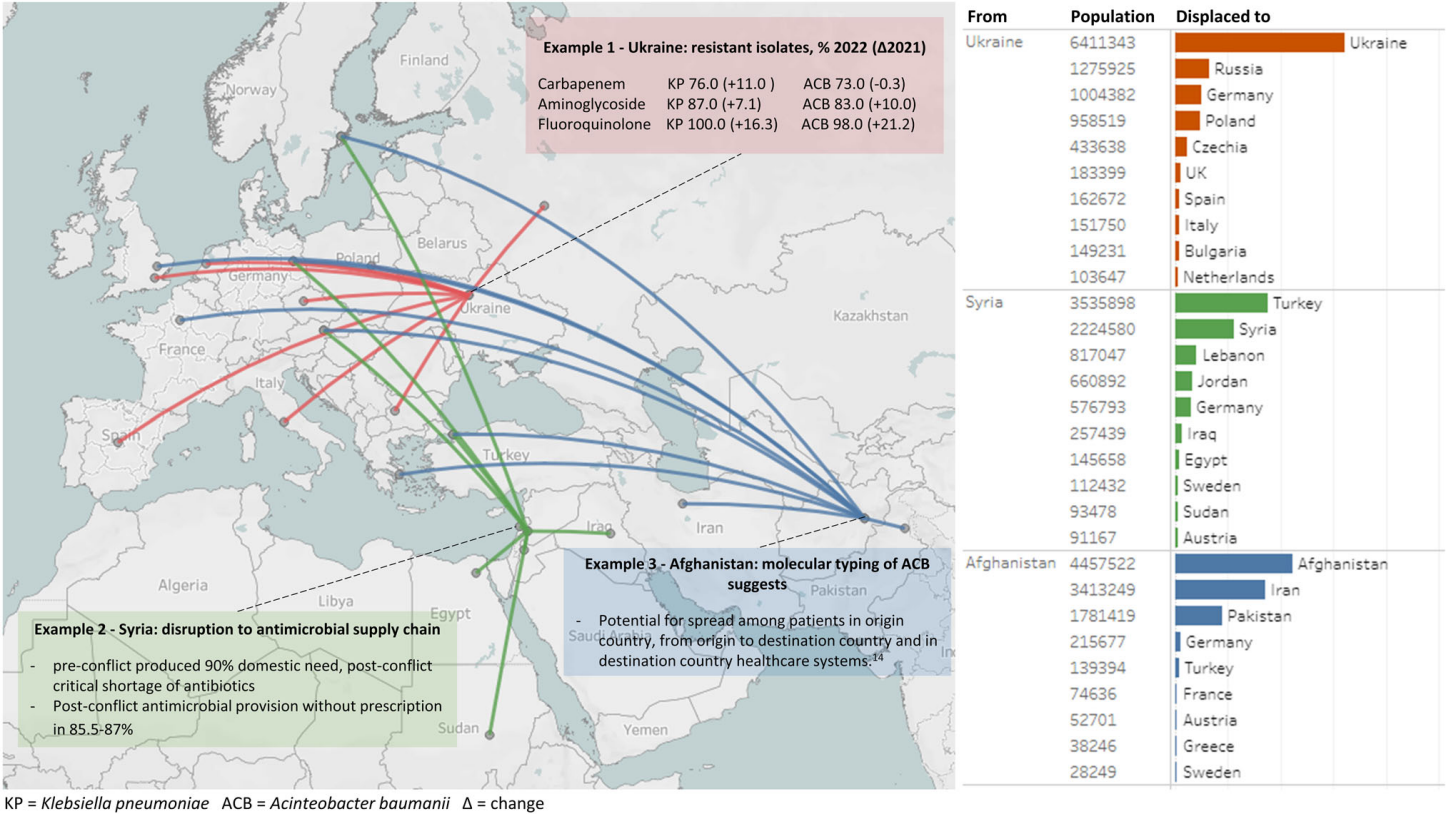
Map showing movement of forcibly displaced peoples during conflicts in Ukraine, Afghanistan and Syria as of 2022 and the associated antimicrobial resistance pressures. The coloured arrows show people predominantly moved to neighbouring countries. Of those displaced from their country of origin and in need of international protection, over half originated from conflicts associated with these three countries^5^. Example 1 (red; Ukraine), the proportion of AMR microbes isolated from patients with infections of war-related injuries increased considerably in 2022 compared to 2021. Data was obtained from nationally collected surveillance data (2021; https://apps.who.int/iris/rest/bitstreams/1496762/retrieve; accessed 07 July 2023) and EUCAST supported sentinel susceptibility testing (2022)^22^ in the context of eruption of major widespread conflict in March 2022 Example 2 (green, Syrian Arab Republic) highlights that availability of antibiotics decreased during the conflict which led to a critical shortage and loss of drug supply security^17,18^. Example 3 (blue; Afghanistan) highlights molecular typing research that showed potential chains of transmission of AMR spread to the destination countries^19^. Panels on the right demonstrate the top 10 destination locations for displaced populations for each example, with the number who moved stated and the proportion received by destination countries in the bar chart.

Conflict results in populations with significant medical needs being forced to travel^5^. The United Nations High Commissioner for Refugees (UNHCR) Global Trends Report for 2022 estimated that almost 110 million people (>1:74 of the global population) had been forcibly displaced within their country of origin or to neighbouring countries globally^5^. This represented an increase of 19 million people compared to 2021, the largest single increase since records began in 1992^5^. Of those displaced to another country and in need of international protection, over half originated from Afghanistan, the Syrian Arab Republic or Ukraine, three countries currently or recently experiencing major human conflict (Fig. 1)^5^. Most displaced people (70%) are hosted in neighbouring countries (Fig. 1)^5^.

## AMR increases in conflict zones

Human conflict has the potential to drastically accelerate the evolution and spread of MDRO, driving AMR globally^6,7^. Damage to infrastructure providing water and sanitation can accelerate AMR development. In addition, damage to laboratory infrastructure, which is already often underdeveloped in many conflict areas, can limit any ongoing testing for microbes (microbiological and antimicrobial susceptibility testing; AST). Disruption to healthcare can disrupt IPC and Public Health activities, such as vaccination programmes^6-8^. High incidence of traumatic bone and soft-tissue injuries in people exposed to the conflict necessitates damage-control surgery, which is often delivered in informal facilities that lack the usual infection controls, resulting in wounds being more easily contaminated with environmental organisms^8,9^. To address these issues, healthcare providers use broad-spectrum antibiotics, which can also increase development of AMR^10^. More recently, conflict-related heavy metal contamination of the environment has been raised as a possible driver for the emergence of novel mechanisms of resistance^7^.

## Multidrug-resistant organisms associated with conflict

Multidrug-resistant (MDR) *Acinetobacter baumanii* complex was recognised as a significant issue among US military personnel following combat-related injuries in Afghanistan and Iraq in 2004 (Fig. 1)^11^. Similarly in the UK, imported MDR *A. baumanii* from Iraq and Afghanistan has been recognised as a potential source of severe nosocomial infection^5,12,13^. The ability of *A. baumanii* to rapidly develop resistance to multiple classes of antibiotics has increased appreciation of its clinical significance in nosocomial infection^8^. Early use of molecular typing methods has demonstrated the potential for clonal outbreaks of MDRO in healthcare settings (Fig. 1) (although it has, as yet, been unable to delineate chains of transmission), with particular risk in intensive care and burns units. There is also the potential to seed plumbing systems, where spread can compromise both individual patient care and operational capability of the service^8,14^.

Military and civilian war casualties transferred from Libya to German hospitals were also observed to be colonised with high rates of MDRO^15^. Among 67 patients transferred during 2016–2017, Methicillin-resistant Staphylococcus aureus (MRSA) and MDROs were observed in 16% and 60% respectively including 37 isolates producing carbapenamases (such as New-Delhi metallo beta-lactamase (NDM): 17, oxacillinase (OXA)−48: 15, OXA-23: 9)^15^. Carbapenamases are β-lactamases, enzymes that often confer resistance to antimicrobial agents, including to the antibiotics that are used when AMR is known to be a problem, known as last line antibiotics^15^. Among Syrian war casualties requiring surgery between 2011–2013, MDROs accounted for 69% surgical site infections, including high rates of MDR *A. baumanii*, extended spectrum beta-lactamase (ESBL)-producing *E.coli* and MRSA^16^. Studies from Syria, neighbouring countries and European hospitals have reported increased incidences of MDRO carriage linked to the Syrian conflict, in particular Enterobacterales producing ESBL-enzymes or NDM-enzymes and MDR *A. baumanii* in 33–83% of individuals^17^. Conflict can also severely limit national and regional antimicrobial supply chains and the capacity to engage in stewardship (an organised programme to promote appropriate use of antimicrobials and preserve their future usefulness). This type of stewardship is often limited or missing even before conflict commenced. Prior to the eruption of conflict, Syria produced 90% of its domestic antibiotic requirement and almost USD200 million from export regionally^18^. Since then, the United Nations has announced critical shortages in antibiotic availability in Syria and studies from 2015 demonstrate the vast majority of antibiotics are now sold within the country without a prescription^18^, further reducing the ability to ensure drug quality or control supply (Fig. 1).

## Recent experience following the war in Ukraine

The conflict currently occurring in Ukraine represents the fastest-growing refugee crisis in Europe since the Second World War (www.unhcr.org/hk/en/73141-ukraine-fastest-growing-refugee-crisis-in-europe-since-wwii.html; accessed 07 July 2023). Prior to the outbreak of widespread conflict in 2022, Ukraine submitted surveillance data to the Central Asian and European Surveillance of AMR network that suggested increasing rates of MDRO (https://apps.who.int/iris/rest/bitstreams/1496762/retrieve; accessed 07 July 2023), particularly among military personnel and likely driven by injudicious antimicrobial use and limited capacity for IPC practices in management of casualties sustained since the annexation of areas of Ukraine by Russia in 2014^19^. Early, limited data suggests the eruption of widespread conflict has considerably exacerbated this issue^20–22^. The Netherlands national MDRO surveillance system has reported 58 patients with recent travel from Ukraine, of which half had recent hospital exposure in Ukraine prior to travel, in striking contrast to their pre-war experience where no MDROs associated with Ukrainian travel had been detected^20^. Increasing numbers of MDR *Klebsiella pneumoniae* isolates that produce NDM-1 or NDM-1/OXA-48 were reported in the Netherlands^20^. This has also been seen in Germany^21^, with whole genome sequencing showing predominance of ST147 and ST307 (successful epidemic clones) disseminated from Ukraine and followed by onward transmission within Germany^21^.

Sentinel testing (random testing to provide data that is potentially representative of the targeted condition at the hospital level) of hospitalised individuals with infected, war-related injuries in Ukraine during 2022 revealed most Gram negative isolates were resistant to the antibiotic meropenem (58%), with *K. pneumoniae* (76%) and *A. baumanii* (73%) producing NDM and OXA-48 carbapenemase enzymes predominating^22^. Of concern, nine (all *K.pneumoniae*) were resistant to all antimicrobials tested^21^. Extended AST demonstrated high rates of resistance to a range of β-lactam β-lactamase inhibitor combinations (often last line antibiotic options for MDROs), including ceftazidime-avibactam, as well as cefiderocol^22^. Clinical outcome data for treating war-related infected injuries is severely limited, but such reports will be important to identify the most suitable antimicrobials for post-injury and peri-operative guidelines^23^.

## Recommendations from prior experience

Effective IPC can be difficult to achieve for populations displaced due to conflict^8^. Implementation of basic practices, including hand hygiene, isolation of patients or keeping patients with the same infections together, have been shown to reduce further spread during conflict-associated *A. baumanii* outbreaks^8^. Strong IPC experience and leadership, strict adherence to basic precautions, and development and implementation of standardised guidelines relevant to the particular context along the entire patient care pathway is essential^8^. This should be provided in parallel to antimicrobial stewardship, supported by adequate diagnostics, to reduce inappropriate use of antibiotics and prevent spread of MDRO^8^, Emergency medical care in these environments is often delivered by an array of organisations ranging from redeployed host country workforce, the United Nations, the World Health Organisation (WHO) or Allied nation militaries to non-governmental organisations such as Médecins Sans Frontières. Active collaboration is needed to optimise provision of expertise and the potential to conduct surveillance to reduce the spread of AMR at each point along the patient pathway.

Given the high rates of MDRO observed in patients from Ukraine, screening and isolation when people from conflict areas are admitted to hospitals in other countries is recommended^20,21^. This seems reasonable pending further characterisation of the risk factors for MDRO carriage among both military and civilian populations, such as prior contact with healthcare services in the conflict area, time since move to alternative country and prior history of conflict-related injury. But care should be taken to explain screening to patients to avoid feelings of discrimination among forcibly displaced and vulnerable populations.

## What else is needed going forward

Currently, reports on conflict-related MDRO incidence from those admitted to European hospitals have been limited by low patient numbers^6,20–22^. A greater understanding of MDRO carriage and infection in both military and civilian peoples following conflict would benefit patient care and outcomes in addition to decreasing the risk of MDRO pathogens spreading unchecked across healthcare facilities globally^23^. Studies of MDRO carriage and infection among the general population and hospitalised patients need to be ambitious to be representative. Such data could inform targeted antimicrobial policy, and limit the need to use empiric broad-spectrum perioperative and point-of-wounding antibiotics^23^. The provision of support to nations experiencing conflict should ideally focus on capacity building to enable sustained capability and delivery of in-country surveillance. In February 2023, the WHO published guidance on methods to optimise nationally representative surveys for those countries with limited surveillance capacity (https://apps.who.int/iris/handle/10665/366150). Adoption of minimal data sets and utilising readily available key resistance indicator antibiotic susceptibility disc testing, could help maximise data representation and highlight isolates for further testing^24^. Where possible, use of molecular assays or bench side tests such as carbapenamase lateral flow immunoassays could then provide valuable insight to prevalent resistance mechanisms. This will be integral to understanding the differential risk across population groups at each point along the patient pathway.

Currently, surveillance strategies for many nations are driven by individual National Action Plans^2^ By necessity, these plans need to be developed, ratified and implemented by individual nations. Severe reduction in capability to conduct AMR surveillance, such as that seen during conflict, compounds a lack of data and further exacerbates the challenges of raising awareness. An additional level of international strategy, specifically aiming to support countries experiencing conflict, would be globally beneficial to reduce AMR burden. This should also involve provision of IPC training, where required, as a key aspect to reduce further spread of conflict-related AMR^25^. This may be best facilitated by regional WHO offices with support from regional public health bodies, such as the European Centre for Disease Prevention and Control.

## Conclusion

Human conflict is an important driver of AMR with consequences for healthcare systems globally. Adherence to IPC precautions at all stages of healthcare contact is key to reducing further spread of MDRO. Surveillance studies are required to inform both risk and appropriate antimicrobial guidelines, but must be ambitious to be representative and will probably require support from neighbouring countries and regional public bodies. The need for such collaboration will require considerable policy development at the international level and, given their role in supporting the development of National Action Plans, may best be coordinated by the Regional Offices of the WHO.
